# Study protocol, rationale and recruitment in a European multi-centre randomized controlled trial to determine the efficacy and safety of azithromycin maintenance therapy for 6 months in primary ciliary dyskinesia

**DOI:** 10.1186/s12890-016-0261-x

**Published:** 2016-07-22

**Authors:** Helene E. Kobbernagel, Frederik F. Buchvald, Eric G. Haarman, Carmen Casaulta, Samuel A. Collins, Claire Hogg, Claudia E. Kuehni, Jane S. Lucas, Heymut Omran, Alexandra L. Quittner, Claudius Werner, Kim G. Nielsen

**Affiliations:** Danish Paediatric Pulmonary Service, Copenhagen University Hospital, Rigshospitalet, Copenhagen, Denmark; Department of Pediatric Pulmonology, VU University Medical Center, Amsterdam, Netherlands; Division of Respiratory Medicine, Department of Pediatrics, Inselspital and University of Bern, Bern, Switzerland; PCD Centre, NIHR Respiratory Biomedical Research Unit and Wellcome Trust Clinical Research Facility, University of Southampton, Southampton, UK; Paediatric Respiratory Department, Royal Brompton Hospital, London, UK; Institute of Social and Preventive Medicine, University of Bern, Bern, Switzerland; Department of General Paediatrics, University Children’s Hospital Muenster, Muenster, Germany; Department of Psychology, University of Miami, Coral Gables, Florida USA

**Keywords:** Primary ciliary dyskinesia, Azithromycin, Lung clearance index, Multiple breath washout, Health-related quality of life, QOL-PCD, Exacerbation

## Abstract

**Background:**

Clinical management of primary ciliary dyskinesia (PCD) respiratory disease is currently based on improving mucociliary clearance and controlling respiratory infections, through the administration of antibiotics. Treatment practices in PCD are largely extrapolated from more common chronic respiratory disorders, particularly cystic fibrosis, but no randomized controlled trials (RCT) have ever evaluated efficacy and safety of any pharmacotherapeutics used in the treatment of PCD. Maintenance therapy, with the macrolide antibiotic azithromycin, is currently widely used in chronic respiratory diseases including PCD. In addition to its antibacterial properties, azithromycin is considered to have beneficial anti-inflammatory and anti-quorum-sensing properties.

The aim of this study is to determine the efficacy of azithromycin maintenance therapy for 6 months on respiratory exacerbations in PCD. The secondary objectives are to evaluate the efficacy of azithromycin on lung function, ventilation inhomogeneity, hearing impairment, and symptoms (respiratory, sinus, ears and hearing) measured on a PCD-specific health-related quality of life instrument, and to assess the safety of azithromycin maintenance therapy in PCD.

**Methods:**

The BESTCILIA trial is a European multi-centre, double-blind, randomized, placebo-controlled, parallel group study. The intervention is tablets of azithromycin 250/500 mg according to body weight or placebo administered three times a week for 6 months. Subjects with a confirmed diagnosis of PCD, age 7–50 years, are eligible for inclusion. Chronic pulmonary infections with Gram-negative bacteria or any recent occurrence of non-tuberculous mycobacteria are exclusion criteria. The planned number of subjects to be included is 125. The trial has been approved by the Research Ethics Committees of the participating institutions.

**Discussion:**

We present a study protocol of an ongoing RCT, evaluating for the first time, the efficacy and safety of a pharmacotherapeutic treatment for patients with PCD. The RCT evaluates azithromycin maintenance therapy, a drug already commonly prescribed in other chronic respiratory disorders. Furthermore, the trial will utilize the Lung clearance index and new, PCD-specific quality of life instruments as outcome measures for PCD. Recruitment is hampered by frequent occurrence of *Pseudomonas aeruginosa* infection, exacerbations at enrolment, and the patients’ perception of disease severity and necessity of additional management and treatment during trial participation.

**Trial registration:**

EudraCT 2013-004664-58 (date of registration: 2014-04-08).

## Background

Primary ciliary dyskinesia (PCD) is a rare, congenital disease with symptom onset in the neonatal period or early childhood, with progression throughout adulthood. PCD presents with chronic rhino-sinusitis, recurrent otitis media and conductive hearing impairment, chronic productive cough and infection and inflammation of the lower respiratory tract [1, 2]. The cilia lining the respiratory epithelium are either immotile or dyskinetic and therefore cannot generate coordinated ciliary beating necessary to expel mucus [3]. The defective mucociliary clearance causes excessive mucus, bacteria and debris to accumulate in the airways, serving as a nidus for infection [4, 5]. Recurrent lower respiratory tract infections progress to chronic infection that leads to bronchiectasis. PCD causes loss of lung function and in severe cases, can result in chronic respiratory failure, with lung transplantation as the final therapeutic avenue [1, 6]. Ciliary beating is important in organ systems other than the respiratory tract such as the embryonic node, sperm flagella, female reproductive tract, and ependyma of the brain and spinal cord [3]. Patients with PCD can therefore also present with situs inversus, reduced fertility, hydrocephalus or, more rarely, complex congenital heart disease. PCD is a genetically heterogeneous disorder, which is predominantly inherited as autosomal recessive trait [1, 7]. Rough estimates of the prevalence of PCD indicate that it affects 1 in 10.000–20.000 individuals [2, 8]. PCD respiratory disease shares some similarities with cystic fibrosis (CF), in which mucociliary clearance is also impaired [9].

As with all chronic respiratory diseases, the aim of treatment for PCD is to maintain or restore normal lung function to the extent possible. The clinical management of PCD respiratory disease is focused on improving mucociliary clearance, primarily with chest physiotherapy, and controlling respiratory infections through the administration of antibiotics [1, 6, 10, 11]. No orphan drugs are available for the treatment of PCD, and the current treatment practices in PCD are extrapolated from treatment of other chronic respiratory disorders, particularly CF, and therefore based on the pathophysiology of these diseases. This strategy is questionable since PCD exhibits a different pathophysiology [1, 12]. No randomized controlled trials (RCT) have been conducted in PCD to determine the efficacy and safety of those pharmacotherapeutics currently used; and thus there are no evidence-based guidelines for pharmacotherapy of PCD patients [1, 11]. Accordingly, management strategies for PCD vary widely between and within the European countries [12]. The lack of evidence for management of PCD respiratory disease is a likely reason for the poor clinical impact on preventing decline in lung function - even when diagnosis is made at an early age [8]. Appropriate treatment of PCD will likely reduce the morbidity of PCD and prolong the life expectancy of PCD-patients.

There is anecdotal evidence of positive outcomes of long-term prophylactic antibiotic treatment in PCD [10, 11]. Over the last decade, maintenance therapy with macrolide antibiotics has been studied in a number of trials in different chronic respiratory diseases, following evidence of effectiveness of low-dose long-term therapy with erythromycin in diffuse panbronchiolitis [13]. In addition to its antibacterial properties, the macrolide antibiotic azithromycin is considered to have beneficial properties with respect to anti-inflammation and quorum sensing inhibition [13–15]. Azithromycin has a simple dosing schedule and covers a wide range of bacteria encountered in PCD respiratory infections. A Cochrane review [16] has reported a meta-analysis evaluating the efficacy and safety of azithromycin maintenance therapy in CF patients. RCTs examining azithromycin versus placebo demonstrated improvement in lung function after a treatment period of 6 months and a significant reduction in the need for additional oral antibiotics in those patients receiving azithromycin. Patients receiving azithromycin were also approximately twice as likely to be free of pulmonary exacerbation at six months [16]. Adverse events were uncommon, although more frequent gastrointestinal events were reported in one trial. Azithromycin maintenance therapy, however, has been associated with an increase in resistance to macrolides [16, 17]. Two RCTs investigating azithromycin maintenance therapy versus placebo in adult patients with non-CF bronchiectasis for 6 and 12 months, respectively, have been published [18, 19] and showed decreases in exacerbations, whereas improvement in lung function, measured by spirometry, and health-related quality of life was only found in the 12 month RCT. Azithromycin maintenance therapy was generally well-tolerated, although gastrointestinal symptoms were reported more frequently in the azithromycin group [18, 19]. Decreases in exacerbations attributable to long-term azithromycin therapy were also demonstrated in a RCT with Indigenous children with non-CF bronchiectasis or chronic suppurative lung disease [20]. Azithromycin maintenance therapy has also been shown to have efficacy in chronic obstructive pulmonary disease. However, hearing decrements were observed to be more common in the azithromycin group in a 12 month RCT in patients with chronic obstructive pulmonary disease [21]. Azithromycin maintenance therapy is currently widely used in the treatment of patients with CF [14, 22] and other chronic respiratory diseases including PCD, however there is no evidence to support its use in PCD.

### Aims

This trial will generate the first evidence-based knowledge on pharmacotherapy in PCD. The main objective is to determine the efficacy of azithromycin maintenance therapy for 6 months on respiratory system exacerbations in subjects with PCD, 7–50 years of age.

The secondary objectives of this trial are to:

- Determine the efficacy of azithromycin on lung function measured by spirometry, on static lung volumes and airways resistance measured by body plethysmography, and on ventilation inhomogeneity measured by nitrogen multiple breath washout (N_2_ MBW) as a new outcome measure;
- Assess the efficacy of azithromycin on respiratory, sinus and ear symptoms as measured by new, PCD-specific health-related quality of life outcome measure (QOL-PCD) [23];
- Determine the efficacy of azithromycin on hearing impairment;
- Determine the efficacy of azithromycin on sputum microbiology and inflammatory markers;
- Assess the safety of azithromycin maintenance therapy in PCD.

This study tests the hypothesis that maintenance therapy with azithromycin provides significant improvement in PCD respiratory disease compared to placebo, by reducing respiratory system exacerbations and by improving lung function, ventilation inhomogeneity, health-related quality of life and hearing impairment. Maintenance therapy with azithromycin is expected to be safe with no serious adverse events compared to placebo.

## Methods/Design

### Study design

**Fig. 1:**
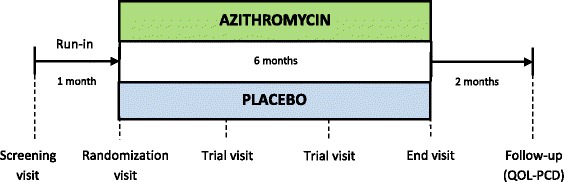
Trial flow chart

This study is a multi-centre, parallel group, double-blind, randomized, placebo-controlled trial with azithromycin maintenance therapy in subjects with PCD (Fig. 1).

### Eligibility

Inclusion criteria for the trial are a confirmed diagnosis of PCD; age 7–50 years; forced expiratory volume in one second (FEV_1_) above 40 % predicted at screening; at least 30 days of treatment with antibiotics prescribed against respiratory tract infections or exacerbations in the previous 2 years; no treatment with azithromycin within 1 month prior to screening; and no current treatment with systemic or inhaled maintenance antibiotics. A confirmed diagnosis of PCD in this trial is based on the following: characteristic clinical symptoms and high speed video microscopic recordings of abnormal ciliary beat pattern and/or frequency, in combination with either an abnormally low nasal NO production <77 nl/min [24], abnormal ciliary ultra-structure demonstrated by transmission electron microscopy analysis or high resolution immunofluorescence (isolated inner dynein arm defects is not accepted), and/or a genetic mutation recognized to cause PCD.

Exclusion criteria are infection with non-tuberculous mycobacteria, *Achromobacter xylosoxidans*, *Burkholderia cepacia* or chronic infection with *Pseudomonas aeruginosa* (chronic infection with *Pseudomonas aeruginosa* is in this trial defined as culture of *Pseudomonas aeruginosa* in at least half of the sputum cultures within the last year provided that at least three sputum cultures are available); allergic reaction to macrolides or any of the ingredients of the azithromycin or placebo tablets; alanine transaminase twice or more the upper limits of normal or history of portal hypertension; serum creatinine above 150 μmol/l or GFR above 50 ml/min; congenital or acquired prolonged QT interval, cardiac arrhythmia, clinical relevant bradycardia, severe heart failure or electrolyte disturbances; myasthenia gravis; current treatment with certain other medicinal products known to possibly interact with azithromycin or to prolong QT interval; pregnancy, breastfeeding or for women use of unreliable forms of contraception; or requirement of home oxygen or assisted ventilation.

### Recruitment

Eligible patients are identified from six European PCD outpatient clinics (see Table 1). Patients and their parents are approached and informed consent obtained.

**Table 1:** Eligible patients and recruitment plans

Trial sites:	Estimated no. of eligible patients:	Recruitment strategies:
Copenhagen University Hospital, Rigshospitalet, Denmark	30	- Patients attending the outpatient clinic.
		- Information at PCD patient event.
University Children's Hospital Muenster, Germany	30	- Patients attending the outpatient clinic.
		- Advertisement via the national PCD patient organization.
		- Information through the German Pediatric Pulmonology Society.
Inselspital Bern, Switzerland	10	- Patients attending the outpatient clinic.
		- Information to patients in the national PCD registry.
VU University Medical Center Amsterdam, Netherlands	20	- Patients attending the outpatient clinic.
		- Advertisement via the national PCD patient organization.
		- Contacting pediatric pulmonologists caring for PCD patients at other academic centers.
University Hospital Southampton, United Kingdom	10	- Patients attending the outpatient clinic.
		- Advertisement via the national PCD patient organization.
Royal Brompton Hospital, United Kingdom	40	- Patients attending the outpatient clinic.
		- Advertisement via the national PCD patient organization.
Total	140	

### Intervention

The intervention is oral tablets of azithromycin or identical tablets of placebo administered three times a week (Monday-Wednesday-Friday) for 6 months. The dose of azithromycin is 250 mg for participants with a body weight below 40 kg at screening and 500 mg for participants weighting 40 kg or above, corresponding to a weekly dose of 750 mg or 1500 mg azithromycin, respectively.

Systemic or inhaled antibiotic treatment, besides the potential azithromycin maintenance therapy during the trial, is only allowed if needed during clinical exacerbations and in the case of new infection with *Pseudomonas aeruginosa*, *Achromobacter xylosoxidans* or *Burkholderia complex*. Treatment of respiratory system exacerbations, if culture and antibiotic susceptibility is not yet known, will be 14 days of oral amoxicillin with clavulanic acid as first-line treatment and clarithromycin as second choice. More severe exacerbations will be treated according to local guidelines. Treatment of first time or intermittent colonization with *Pseudomonas aeruginosa* will be inhalation of colistin in combination with oral ciprofloxacin or inhalation of Tobramycin for 3-4 weeks. In case of treatment failure, early relapse or signs of development of chronic *Pseudomonas aeruginosa* infection, antibiotic treatment will be intensified to last for 3 months. Treatment with the trial medication will continue throughout the trial simultaneously with any antibiotics prescribed for exacerbations or infection with *Pseudomonas aeruginosa*. Any other pre-trial medication will continue unchanged, and any other new prescriptions, which are considered necessary for the participant’s welfare, may be given.

### Randomization, allocation and blinding

Participants will be randomly assigned to one of the two treatment arms in a 1:1 ratio with stratification according to age (7–12 years; 13–21 years; and 22–50 years). The allocation sequence is a permuted-block randomization and has been generated by an external consultancy (Datamanagement, Public Health and Quality Improvement, Central Denmark Region, Aarhus, Denmark). Participants will be assigned to the intervention by delegated study staff via an online randomization module in an electronic Case Report Form (‘TrialPartner’, Datamanagement, Denmark). The allocation is concealed to the investigative staff and sponsor throughout the study, and unblinding will only occur in the event of a Suspected Unexpected Serious Adverse Reaction or if otherwise required for patient safety.

A hospital pharmacy (Apotheek Haagse Ziekenhuizen, The Hague, The Netherlands) is responsible for preparation of the trial medication. Azithromycin tablets bought from a pharmaceutical company are repackaged and identical placebo tablets are specifically manufactured by the hospital pharmacy. Blinded labelling and packaging of the trial medication in identical blister packages in carton boxes is performed centrally at the hospital pharmacy according to Good Manufacturing Practice. Adherence to the trial treatment will be assessed by participant/parent report and count of returned trial medications.

### Data collection

Participants will be screened for eligibility in a stable state after having given informed consent to participation. At screening, demographic information (age, gender, ethnicity) will be recorded and inclusion- and exclusion criteria review will be performed, including data on medical history, current symptoms and concomitant medication, physical examination, pregnancy test, electrocardiogram, spirometry, and sputum microbiology. The QOL-PCD [23] will be completed by participants both one week prior to screening and again at the screening visit to obtain baseline values. Screening will be followed by a run-in period of one month to ensure that participants have stable lung function (defined as maximal decrease in FEV_1_% predicted of 10 percentage points from the screening visit to the randomization visit) and that prohibited medications are washed out at randomization. Participants will be withdrawn prior to randomization if they have an exacerbation or receive systemic or inhaled antibiotics at the screening or randomization visit, or if they have received antibiotics for more than 2 weeks during the run-in period. Participants will be reviewed every two months during the 6 months treatment period. Participants will be asked to contact the trial site at the onset of an exacerbation, if possible, and exacerbations will be reviewed at extra visits or by telephone contact. Withdrawal of participants during the trial will occur if the participants fulfil any of the exclusion criteria or due to safety reasons. A QOL-PCD will be completed by the participants, as a follow-up assessment 2 months after the end of the treatment period.

All data will be recorded in an electronic case report form. The primary and secondary outcome measures (see below) are collected at the specified time points. All adverse events occurring from the first administration of the trial medication to the end of the last trial visit will be recorded and monitored (Table 2).

**Table 2:** Time and events schedule

	Screening	Treatment period				Follow-up
	Visit *0 months*	Visit *1 month (+2 weeks)*	Visit *3 months (+/- 1 week)*	Visit *5 months (+/- 1 week)*	Visit *7 months (+/- 1 week)*	*9 months*
Informed consent	X					
Demographics	X					
Inclusion/Exclusion criteria review	X					
INTERVENTION:						
Randomization		X				
Azithromycin/Placebo						
Adherence			X	X	X	
ASSESSMENTS:						
QOL-PCD	X	X	X	X	X	X
Patient interview	X	X	X	X	X	
Concomitant medication	X	X	X	X	X	
Weekly patient diary		X	X	X	X	
Physical examination & vital signs	X	X	X	X	X	
Pregnancy test	X	X	X	X	X	
Electrocardiogram	X					
Spirometry	X	X	X	X	X	
Body plethysmography		X	X	X	X	
N_2_ MBW		X	X	X	X	
Audiometry & tympanometry		X			X	
Sputum microbiology & antibiotic susceptibility	X	X	X	X	X	
Inflammatory markers (sputum & blood)		X			X	
Blood samples (bone marrow, liver & kidney)		X			X	

### Outcomes

#### Primary outcome

##### Number of exacerbations

The primary outcome is the number of respiratory system exacerbations occurring during the treatment period. A respiratory system exacerbation is defined in this trial as either respiratory tract symptoms leading to start of systemic antibiotic treatment, irrespective of results of bacterial culture, or as a decline in FEV_1_% predicted equal to or above 10 percentage points relative to the average of FEV_1_% predicted at screening and randomization, whether or not antibiotics are prescribed.

The occurrence of exacerbations will be assessed by patient interview, physical examination and spirometry. At each trial visit, and at any extra contacts with the trial sites attributable to exacerbations, the participants will be interviewed regarding symptoms and concomitant medication since last contact with the trial site. The interview will be supplemented by a weekly patient diary on symptoms and antibiotics. A physical examination reviewing the participants’ general condition, vital signs, ears, heart and lungs will be performed at all visits.

### Secondary outcomes

#### Lung function measured by spirometry

Spirometry will be performed according to the American Thoracic Society/European Respiratory Society (ATS/ERS) criteria [25] at each visit. The outcomes from spirometry are FEV_1_, forced vital capacity (FVC) and forced expiratory flow at 25–75 % of FVC with results expressed in percentage of predicted [26]. Short and long acting bronchodilators and anticholinergics will be withheld 6 and 12 h prior to spirometry, respectively.

#### Static lung volumes & airways resistance

Measurement of residual volume (RV)% predicted, RV/total lung capacity (TLC)% predicted and airway resistance (Raw)% predicted by body plethysmography will be performed at all trial visits during the 6 month treatment period using a standardised whole-body plethysmograph and according to ATS/ERS recommendations [27].

#### Ventilation distribution inhomogeneity

Assessment of ventilation distribution inhomogeneity by nitrogen multiple breath washout (N_2_ MBW) will be performed at all the trial visits during the treatment period. The outcomes derived from N_2_MBW will be lung clearance index (LCI), which is a measurement of overall ventilation inhomogeneity in the lungs, and the indexes S_acin_xV_T_ and S_cond_xV_T_ reflecting ventilation inhomogeneity in the small conducting and acinar airways, respectively. N_2_ MBW will be measured according to ERS/ATS Consensus Statement for inert gas washout measurement [28] and will be performed prior to spirometry. All trial sites will use the instrument EXHALYZER®D (ECO MEDICS AG, Duernten, Switzerland) to perform N_2_ MBW, and results of the N_2_ MBW will be analysed using the software ‘Spiroware’ (ECO MEDICS AG, Duernten, Switzerland).

#### Health-related quality of life

Newly developed, PCD-specific health-related quality of life questionnaires (QOL-PCD) will be used to assess patient-reported outcomes [23]. The QOL-PCD has age-appropriate versions available for children 6–12 years, adolescents 13–17 years, adults, and a parent proxy. The following domains on the QOL-PCD will be used as outcome measures: Respiratory Symptoms, Sinus Symptoms, and Ears and Hearing. The QOL-PCD is available in the primary languages of the participating countries (English, Danish, Dutch, German and Swiss German), and an electronic speaking questionnaire is available for the youngest age group. The QOL-PCD will be completed at all visits, at extra contacts with trial sites attributable to exacerbations, and will be administered prior to any other study procedures to reduce bias.

#### Hearing impairment

Audiometry and tympanometry will be performed at randomization and at the end of the treatment period at local hospital departments of otorhinolaryngology. Hearing thresholds will be determined as the pure tone average at 0.5, 1, 2, 4 & 8 kHz measured by air conduction and as discrimination loss. Bone conduction thresholds will only be measured if air conduction thresholds are above 10 dB.

#### Sputum microbiology

Sputum will be sampled at all visits for culture of bacteria and fungi. When the participant cannot cough up sputum voluntarily, then sampling of sputum is performed according to local procedures e.g. by use of cough swabs or by laryngeal suction. Sputum will be cultured for at least the following microorganisms: *Streptococcus pneumonia*, *Group A beta-haemolytic Streptococcus*, *Staphylococcus aureus*, *Moraxella catharrhalis*, *Haemophilus influenzae*, *Pseudomonas aeruginosa*, *Burkholderia cepacia complex*, *Achromobacter xylosoxidans* and *Aspergillus fumigatus*. Microscopy of bacteria will not be performed. During an exacerbation, participants will be asked to deliver sputum samples, which, in addition to regular cultures, will be analysed for respiratory viruses and mycoplasma pneumoniae by polymerase chain reaction. The testing will include the following viruses: Influenza A and B, rhino virus, adenovirus, respiratory syncytial virus, bocavirus, enterovirus, metapneumovirus and parainfluenza virus. Culture incl. antibiotic susceptibility testing and polymerase chain reaction are performed at the local microbiological laboratories.

#### Inflammatory markers

In those participants who can expectorate sputum and in participants where laryngeal suction is performed, a sample of sputum from randomization and from the end of the treatment period will be stored in a biobank for later analysis of cytokines and neutrophil elastase. Blood samples will be taken at simultaneous time points for measurement of C-reactive protein, white cell count including differential cell counts and cytokines. C-reactive protein and white cell count including differential counts will be analysed in the local hospital laboratories. The blood samples for measurement of cytokines will be centrifuged at the local hospital laboratories to isolate the serum, and the serum will be kept in a biobank for later analysis. After the end of the trial, all the serum samples and sputum samples will be analysed for cytokines and neutrophil elastase collectively at the Department of Clinical Microbiology at Rigshospitalet in Denmark.

#### Adverse events

Participants will be assessed for the occurrence of adverse events using the following measures: Interview regarding any new or worsening of symptoms; physical examination including vital signs; microbiological analysis of sputum and antibiotic susceptibility testing; audiometry; and blood samples to monitor bone marrow, liver and kidney function. Interview, physical examination, and culture of sputum and antibiotic susceptibility testing will be performed at all visits, whereas audiometry and blood sampling will only be performed at randomization and at the end of the treatment period. At screening and end of the treatment period, sputum will be tested for non-tuberculous mycobacteria [29, 30]. The antibiotic susceptibility testing will at least include susceptibility of bacteria to azithromycin and either amoxicillin with clavulanic acid or penicillin (depending on the bacterial species). The blood samples will include determination of haemoglobin, white cell count, thrombocytes, alanine transaminase and creatinine and will be analysed in the local hospital laboratories.

An independent data and safety monitoring board will monitor patient safety and treatment efficacy data while the trial in ongoing.

### Sample size

The planned number of participants to be enrolled in the trial is 125. The sample size is calculated based on the primary endpoint and has been estimated based upon detecting a 50 % reduction in number of exacerbations (assuming a mean of 2.5 exacerbations per year in the placebo group) in the azithromycin group with a statistical power of 90 % and assuming Poisson distribution. On this basis, a sample size of 100 participants completing the trial will be required, with 50 participants in each treatment arm. Allowing for a dropout rate of 20 %, the total number of participants to be enrolled in the trial is 125. Power is 70 % to detect a difference of 5 percentage points between treatments in the pre- to post-intervention change in FEV_1_ % predicted (assuming a SD of 10 % for intra-subject change); and power is 88 % to detect a difference of 5 percentage points in the pre- to post-intervention change in LCI (assuming a SD of 8 % for intra-subject change). Calculations of power are based on two-sided testing with an alpha level of 0.05.

### Statistical analysis

Two analysis populations are defined: Intention-to-treat population and per-protocol population. All participants who are randomized will be included in the intention-to-treat population analysis, and all available data from drop-outs will be included in this intention-to-treat analysis. The per-protocol population analysis will include all participants, who are randomized, have completed the trial, have not been discontinued from treatment, and have had no major protocol deviations. A participant will be considered to have completed the trial if he or she has completed all the five visits. The per-protocol population will be determined prior to unblinding.

Data will be summarized descriptively. For continuous variables, descriptive statistics such as mean and SD, or median and range (depending on normality) will be summarised. For categorical variables, the frequency will be presented. Analysis of the primary and secondary outcomes will be based on the intention-to-treat population and will be repeated based on the per-protocol population. The primary outcome will be analysed using Poisson regression, with adjustment for age as a potential confounder. The continuous secondary outcomes will be modelled using a linear mixed model including a fixed time-specific treatment effect and random effects of trial site and person. Additional potential confounders, such as age, can be adjusted by including them in the linear mixed model as fixed main effects. The categorical secondary outcomes will be analysed by McNemar’s test or test of marginal homogeneity, as appropriate. All statistical inference tests will be performed using two-sided testing with a significance level (alpha) of 0.05. No adjustment will be made to the significance level for the multiple statistical comparisons planned as part of the secondary analyses.

## Discussion

This RCT will be the first to evaluate the efficacy and safety of a commonly prescribed maintenance therapy in PCD, with the potential to improve the clinical management of PCD by providing the first evidence base for pharmacotherapy in this lifelong disease. PCD differs pathophysiologically and clinically from CF and non-CF bronchiectasis and therefore, evidence-based guidelines on pharmacotherapy specifically for PCD are needed. Besides the burden of recurrent pulmonary infections requiring antibiotic therapy, PCD patients suffer from chronic daily symptoms of the upper respiratory tract, including the ears, causing morbidity in everyday life [2] and potentially interfering with social life and educational opportunities [23]. Maintenance therapy with potential efficacy for both upper and lower respiratory tract morbidities is therefore considered highly relevant for PCD. Azithromycin maintenance therapy is chosen for this first RCT on pharmacotherapy for PCD because of its potential anti-inflammatory and antibacterial benefits [13, 15], and its mild side effect profile documented in previous RCTs of azithromycin maintenance therapy [16–20]. Additionally, azithromycin is an easily administered and inexpensive therapy compared to other candidate drugs.

A secondary focus of this trial is the development and evaluation of new outcome measures in PCD. Since the treatment intervention in this RCT is directed against most of the central symptoms of PCD (infections and impairment in the lungs, sinuses and ears), outcome measures will be designed to assess several domains of functioning: respiratory system exacerbations, pulmonary function, health-related quality of life, and hearing impairment.

The number of respiratory system exacerbations is chosen as the primary outcome of the trial because it clearly interrupts normal, everyday activities, requires additional treatment, and may cause irreversible lung damage. No consensus definition of exacerbations in PCD exists, and therefore our definition is based on clinical and patient relevance and feasibility and utility in clinical practise. Pulmonary function will be assessed by both traditional spirometry, body plethysmography and by N_2_ MBW. RV and RV/TLC ratio measured by body plethysmography have been shown to correlate with severity of the total lung impairment on chest high-resolution computed tomography (HRCT) in PCD and has been shown to better predict abnormalities on HRCT scans than spirometric measures [31]. Assessment of ventilation inhomogeneity by MBW has demonstrated abnormal values in PCD, even in patients with FEV_1_ within the normal range, reflecting small airway disease [32–34], and one study [34] has shown LCI measured by N_2_ MBW to be more sensitive than FEV_1_ in detecting PCD patients with structural abnormalities, but there are, however, contradictory findings on whether LCI correlates with HRCT scores in PCD [34, 35]. MBW indexes may prove to be more valuable outcome measures in PCD than the rather insensitive spirometric parameters. LCI is increasingly being used as outcome measure in CF and has been shown to have potential as outcome measure in mild CF lung disease [36, 37].

Finally, we developed patient-reported outcome measures (PROs) for children, adolescents and adults with PCD and parent caregivers. PROs are now accepted by both the FDA and EMA as primary or secondary endpoints in clinical trials and provide unique information on how new medications and treatments affect daily symptoms and functioning [38, 39]. These measures assess several domains of health-related quality of life, including respiratory and sinus symptoms, physical and social functioning, and the perceived burden of treatment. PROs have now been routinely incorporated into randomized trials of new medications in CF and non-CF bronchiectasis, providing critical information about efficacy and impact on daily functioning [40, 41]. This will be the first trial to utilize these new PCD-specific PROs.

Hearing impairment will be evaluated by audiometry and tympanometry partly as an outcome measure for the efficacy of azithromycin maintenance therapy and simultaneously also as a safety measure due to reports of ototoxicity as an adverse reaction of azithromycin [21, 42]. Besides hearing impairment, emergence of antibiotic resistance will also be monitored in the trial as this has been raised as a concern after previous RCTs with azithromycin maintenance therapy showed increased resistance among common respiratory pathogens [18–20, 30].

The challenges of this study may be the wide age range which includes both paediatric and adult PCD patients, and differences in routine management between the trial sites. The trial is designed as a multi-centre study with participation in several European countries due to the rarity of PCD. However, even with recruitment at six trial sites, distributed across five European countries, it is still necessary to include a wide age range to recruit enough patients. With regard to the challenge of differences in routine management between the trial sites, then this is reduced by standardising part of the antibiotic treatment regimen per trial protocol and by excluding PCD patients with chronic *Pseudomonas aeruginosa* from participation in the RCT. The decision to exclude patients with chronic *Pseudomonas aeruginosa* infection from the trial is based on the great variation in treatment practises of *Pseudomonas aeruginosa* between the participating trial sites and because chronic infection introduces a high level of treatment activity, with high dose systemic antibiotic treatment being the most critical confounder.

The RCT is currently ongoing and participants have been enrolled at all trial sites. Recruitment is hampered by our choice to exclude PCD patients with chronic *Pseudomonas aeruginosa* infection and patients receiving systemic or inhaled maintenance antibiotics to limit the impact of concomitant antibiotic therapy. *Pseudomonas aeruginosa* infection has proved to be more common in PCD than previously anticipated [5]. PCD patients without chronic infection are not used to this frequent and time consuming follow-up, and, in contrast to CF patients, PCD patients are in general less well organised in patient organizations, which seem to influence their interest in sacrificing time and burden in research (see Table 3 for reasons for recruitment issues). Scarce knowledge on prognosis and clinical sub-phenotypes in PCD among both physicians and patients probably affects the perception of disease severity. PCD patients and physicians may have different views on the appropriate extent of management and treatment, which is important to take into consideration when designing future trials in PCD. Another limiting factor for inclusion in this trial is the requirement of clinical stability at enrolment, which is challenging in PCD characterized by frequent respiratory exacerbations and a chronic, but somewhat fluctuating degree of respiratory symptoms. Initially, the protocol did not allow any change in medication from the screening visit and throughout the run-in period lasting up to 1½ month. However, we have been forced to allow some oral antibiotics during this period. The fluctuating nature of the PCD disease and the considerable number of patients being on various more or less permanent antibiotic regimens should be carefully considered and weighed against the aims of including only stable and comparable patients, when designing clinical trials in the future.

**Table 3:** Reasons for recruitment issues

Major reasons for refusal of participation:	
Due to patients’ views:	
	- Long travel distance to trial site.
	- Time consuming (e.g. not possible to take time off work).
	- Fear of adverse reactions.
	- Treatment burden.
	- Not perceiving themselves/their child as being ill and therefore finding medical maintenance therapy unnecessary.
	- Not seeing any personal gain in taking part in the research.
	- Already on maintenance antibiotics and not willing to discontinue this treatment.
	- Fear of blood sampling.
Due to eligibility criteria in study protocol:	
	- Chronic *Pseudomonas aeruginosa* infection.
	- Systemic or inhaled maintenance antibiotics due to intermittent *Pseudomonas aeruginosa* infection or patient’s clinical status, which is not considered appropriate to discontinue.
	- Prohibited concomitant medication e.g. antidepressants.
	- Age outside the age range.

An issue worth considering in the planning of investigator initiated trials in rare diseases in Europe is the considerable and time consuming administrative tasks in conducting European multi-centre trials due to the variations in application procedures and reporting to the local authorities, especially the Research Ethics Committees, of the various European countries, which indeed does not seem harmonized in the European Member States despite the EU Clinical Trials Directive (2001/20/EC) [43] and the Voluntary Harmonisation Procedure [44]. Application for administrative support is recommended.

In conclusion, the completion of this RCT will be the first step towards evidence-based guidelines on pharmacotherapy in PCD. Furthermore, the trial will assess the potential of N_2_ MBW indexes and newly developed PCD-specific health-related quality of life questionnaires as outcome measures in PCD. This RCT will thereby not only answer the specific research question on the efficacy and safety of azithromycin maintenance therapy in PCD, but also facilitate future RCTs in PCD by better developing and evaluating appropriate outcome measures.

## Abbreviations

ATS/ERS, American Thoracic Society/European Respiratory Society; CF, cystic fibrosis; FEV_1_, forced expiratory volume in one second; FVC, forced vital capacity; HRCT, high-resolution computed tomography; LCI, lung clearance index; N_2_ MBW, nitrogen multiple breath washout; PCD, primary ciliary dyskinesia; QOL-PCD, quality of life – primary ciliary dyskinesia; RCT, randomized controlled trial; RV, residual volume; SD, standard deviation; TLC, total lung capacity
